# Stereoselective Toxicokinetic and Distribution Study on the Hexaconazole Enantiomers in Mice

**DOI:** 10.3390/toxics11020145

**Published:** 2023-02-02

**Authors:** Guofei Luo, Junxiao Pang, Dali Sun, Qinghai Zhang

**Affiliations:** 1 The Key Laboratory of Environmental Pollution Monitoring and Disease Control, Ministry of Education, School of Public Health, Guizhou Medical University, Guiyang 550025, China; 2 Food and Pharmaceutical Engineering Institute, Guiyang University, Guiyang 550005, China

**Keywords:** hexaconazole, toxicokinetic, stereoselectivity, molecular docking

## Abstract

Hexaconazole (Hex) has been widely used in agricultural products, and its residues may pose a potential risk to human health. However, the metabolic behavior of Hex enantiomers in mammal organisms is still unknown, which is important for evaluating the differences in their toxicity. In this study, the distribution of S-(+)- and R-(−)-Hex in mice was detected by an ultra-high performance liquid chromatography coupled with tandem mass spectrometry (UPLC–MS/MS), and the mechanism differences in the toxicokinetic behavior were analyzed by molecular docking. Good linearities, accuracies, and precisions were achieved for S-(+)- and R-(−)-Hex, with recoveries of 88.7~104.2% and RSDs less than 9.45% in nine tissues of mice. This established method was then used to detect the toxicokinetic of Hex enantiomers in mice after oral administration within 96 h. The results showed that the half-lives of S-(+)- and R-(−)-Hex were 3.07 and 3.71 h in plasma. Hex was mainly accumulated in the liver, followed by the kidneys, brain, lungs, spleen, and heart. The enantiomeric fraction (EF) values of Hex enantiomers in most of the samples were below 1, indicating that S-(+)-Hex decreased faster than its antipode. The molecular docking showed that the binding of S-(+)-Hex with P450arom was much more stable than R-(−)-Hex, which verified the fact that S-(+)-Hex was prefer to decrease in most of the tissues. The results of this study could be helpful for further evaluating the potential toxic risk of Hex enantiomers and for the development and usage of its pure monomer.

## 1. Introduction

Hexaconazole (Hex), a triazole fungicide, has been widely used in rice, fruits, peanuts, and other plants to control rice sheath blight, wheat powdery mildew, apple Alternaria leaf spot, etc., due to its broad spectrum and high efficiency. The mechanism is to destroy or prevent the biosynthesis of ergosterol, causing the failure of cell membrane formation and leading to the death of pathogenic microorganisms [1]. At present, research on Hex has been mainly focused on its analysis method development, residual behavior, toxicity effect, and health risk to the non-target organisms. The environmental behavior of Hex has been studied in mango [2], grape [3], potato [4], rice, soil, and water [5] with gas chromatography (GC), liquid chromatography (HPLC), and liquid chromatography coupled with mass spectrometry (LC–MS/MS). The health risk of Hex in black tea has been evaluated, which showed that the concentration of Hex increased during the process of fermentation, while its risk to humans was relatively low [6]. In the environment, Hex can permeate into groundwater after application, which may pose potential toxic effects in benthonic animals [7]. It was reported that Hex can cause severe oxidative stress on earthworms and interfere in steroid biosynthesis, arachidonic acid metabolism, and the cell cycle [8]. Moreover, Hex showed toxicity to aquatic invertebrates, causing liver damage and reproductive toxicity to zebrafish and disturbing the energy metabolism, lipid metabolism, and amino acid metabolism [9]. 

**Figure 1:**
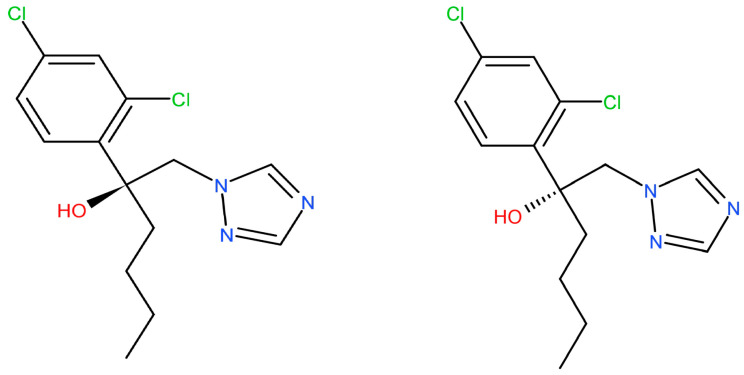
The chemical structures of Hex enantiomers.

Hex has a chiral center with two stereoisomers (Figure 1). The enantiomers exhibit enantioselectivity in the fields of environmental behavior, biological activities, toxic effects, and potential risks [10]. Li et al. reported that S-(+)-Hex decreased faster than its antipode in cucumber and tomato while slower in soil samples [11]. Other reports showed that obviously enantioselective dissipation had been observed with (−)-Hex degrading faster in tomato, enriching its (+)-form [12]. The toxicity effects that were caused by Hex enantiomers differed or were even completely opposite. S-(+)-Hex had higher acute toxicity in earthworms and achieved more serious effects on the contents of malonaldehyde and the activity of cytochrome P450. Moreover, S-(+)-Hex had a higher influence on steroid biosynthesis, arachidonic acid metabolism, and cell cycle processes than R-(−)-Hex [8]. Shen et al. reported that the degradation of Hex enantiomers was gender dependent. (+)-Hex decreased faster than (−)-Hex in male and female rats, while both (+)-Hex and (−)-Hex decreased faster in male rats than in female rats [13]. R-(−)-Hex achieved a higher effect on energy metabolism, while S-(+)-Hex had a more serious influence on amino acid metabolism in zebrafish [9]. The liver microsomal enzymes of mice decreased S-(+)-Hex faster than its antipode [14]. R-(−)-Hex accumulated in zebrafish much more easily than its (+)-enantiomer at the concentrations of 100 and 200 μg/L [15]. Therefore, due to its different behavior in terms of environmental and toxicity effects, Hex should be studied at enantiomer levels.

Toxicokinetics provides imperative information concerning the absorption, distribution, metabolism, and excretion (ADME) process in model animals and the dose-time relationships of the toxicants [16]. The ADME process can be used to monitor the regularity of the occurrence and behavior of toxicants, and provide a scientific basis for the safety evaluation. Jeong et al. studied the toxicokinetics of diisobutyl phthalate and its major metabolite monoisobutyl phthalate in rats by using the UPLC–MS/MS method. A wide distribution, short half-life, and high clearance rate were observed after oral and intravenous administration [17,18]. The toxicokinetic results of amphotericin B liposomes in rats showed this compound was mainly distributed in the liver and kidney, with female rats achieving a higher enrichment ratio, longer half-life, and lower plasma clearance rate than males [19]. However, thus far, research regarding the distribution and behavior of Hex in vivo are rare, and the stereoselectivity in toxicokinetic studies of its enantiomers in mammals is still unclear.

Hex has been reported as an aromatase (P450arom) inhibitor. Aromatase belongs to the cytochrome P450 family, which can catalyze androgen (androstenedione or testosterone) to estrogens (estrone or estradiol) [20]. Trosken et al. demonstrated that Hex could inhibit the activity of P450arom with an inhibition rate (IC_50_) of 35 μM [21]. The technology of molecular docking can reveal the mode of interaction between chemical micromolecules and targeted protein macromolecules, providing information on their structure–activity relationships. Until now, the molecular mechanism of the differences in toxicity and metabolic behavior induced by Hex enantiomers is still unclear.

Therefore, in this study, a UPLC–MS/MS method was established to analyze the toxicokinetics of Hex enantiomers in plasma, urine, feces, and six tissues in mice after a single oral administration, and then the EF values were introduced to evaluate the differences between the two enantiomers in metabolic behavior. Finally, molecular docking was used to explore the interaction between Hex and P450arom to mechanistically clarify the differences caused by enantiomers. The results of this study could be of great importance in making clear the metabolic mechanism of Hex in vivo, and be helpful for its potential risk evaluation at the enantiomer level. 

## 2. Materials and Methods

### 2.1. Chemicals and Reagents

The standard of racemic Hex (purity > 99.0%) was purchased from Dr. Ehrenstorfer GmbH Co., Ltd (Augsburg, Germany). Standards of Hex enantiomers S-(+)-Hex and R-(−)-Hex with a purity of more than 99.0% were provided by Chiralway Biotech Co., Ltd (Shanghai, China). HPLC-grades of acetonitrile (ACN) and methanol, C18, and N-propyl ethylenediamine (PSA) were obtained from Anpel Technologies Co., Ltd., (Shanghai, China). The acetic acid, anhydrous magnesium sulfate (MgSO_4_), sodium chloride (NaCl), and dimethyl sulfoxide (DMSO) of analytical grade were purchased from Kemiou Co., Ltd. (Tianjin, China). Ultrapure water was obtained from Milli-Q water system.

### 2.2. Animal Experimental Design

The animal experiment was carried out strictly according to the OECD guidelines and the ethics requirements authorized by the animal ethical committee. Male Kunming (KM) mice (20–22 g) of SPF grade were purchased from Liaoning Changsheng Biotechnology Co., Ltd. (Liaoning, China) [No. SCXK (Liao) 2015-0001]. The mice were fed in an animal center at a temperature of 25 ± 4 °C and humidity of 50–60% with 12 h light-dark cycles. They were allowed to freely take feedstuff and water for 1 week and were then used for the experiment.

Thirty-six mice were randomly divided into 12 groups, including blank and 11 Hex-treated groups with three mice in each group. After 12 h fasting, 0.2 mL of 0.2 mg L^−1^ racemic mixture of Hex (Rac-Hex) standard solution, which was dissolved in DMSO solution, was injected into the 11 treated groups through oral administration. The blank group was given the same amount of DMSO solution. The mice were sacrificed after 0.5, 1, 2, 4, 6, 8, 10, 12, 24, 48, and 96 h of treatment. Urine and feces samples were collected during the exposure period. Plasma samples were obtained through the orbital sinus and then collected in anticoagulant tubes. They were then centrifuged at 3000 rpm for 10 min, and the serum was collected. After that, the mice were dissected and samples of the heart, lung, liver, kidney, brain, and spleen were collected, weighed, and stored at −80 °C until analysis. 

### 2.3. Sample Preparation

Samples of plasma, urine, and feces of 0.5 g or 0.5 mL were weighed. Six tissue samples were cut into pieces by scissors, ground in a mortar under liquid nitrogen, and weighed for 0.5 g each. Samples were placed into a 5-mL enzyme-free centrifuge tube and added to with 1.5 mL acetonitrile/water mixture (acetonitrile: water = 4:1, *v/v*). After that, 0.3 g NaCl was added to each tube. The samples were then vortexed for 1 min, sonicated for 15 min, and centrifuged at 3000 rpm for 5 min. The supernatant (1 mL) was transferred into 2-mL centrifuge tubes in which 50 mg PSA, 50 mg C18, and 150 mg MgSO_4_ had been added first. They were vortexed for 1 min and centrifuged at 3000 rpm for 5 min. The supernatant was filtered through a 0.22 mm PTFE membrane and placed in a 2-mL injection vial for analysis. 

### 2.4. UPLC–MS/MS Analysis

The determination of Hex enantiomers was performed on a Pekin Elmer 3Q Sight 210 series triple quadrupole mass spectrometer equipped with an electrospray ionization (ESI) ion source (Pekin Elmer, Waltham, MA, USA). The separation of Rac-Hex was carried out on a Lux Cellulose-2 chiral column (250 × 4.6 mm, 3 µm, Phenomenex, Los Angeles, CA, USA) at the temperature of 40 °C. The autosampler tray was maintained at 4 °C. The mobile phase was set as 0.1% formic acid aqueous solution (A) and acetonitrile (B) at the ratio of 3:7 (*v/v*). The flow rate was set as 0.5 mL/min with the injection of 10 µL. The mass spectrometer conditions were set as follows: ion source temperature 400 °C in positive mode (ESI^+^), ion spray voltage 5500 V, curtain and collision gas (nitrogen) 25 and 3 psi, delustering potential voltage (DP) 90 V, and collision energy (CE) 30 V. The precursor ion was transitioned from *m/z* 314.1 to 70.1 (quantitative ion) and from *m/z* 314.1 to 159.0 (qualitative ion). The chromatography data were processed by a 3Q Sight workstation (Pekin Elmer, Waltham, MA, USA).

### 2.5. Method Validation

Detailed information about the standard curve preparation, matrix standard curve preparation, recovery, matrix effects, accuracy and precision, the limit of detection (LOD) and limit of quantification (LOQ), and quality control (QC) are presented in the Supplementary Information.

### 2.6. Enantioselective Toxicokinetic Study

The values of the enantiomeric fraction (EF) were used to detect the enantioselective behavior of the Hex enantiomers in the mice, which are calculated by the following equation:

EF = Concentration of S-(+)-Hex/Concentration of R-(−)-Hex(1)

The EF values ranged from 0 to 1. An EF > 1 indicates S-(+)-Hex-enriched, while an EF < 1 illustrates R-(−)-Hex-enriched.

A two-compartment model was used to analyze the toxicokinetics in plasma, which is shown in Figure S1. The main toxicokinetic parameters in the plasma included the area under the concentration–time curve (AUC), clearance rate (CL), half-life (t_1/2_), peak concentration (C_max_), peak time (t_max_), and apparent volume of distribution (V_d_). These parameters were calculated by the following equations:

∆AUC = (C_i-1_ − C_i_)/k_i_ (2)

AUC_0-∞_ = ∑i = 1^n(C_i-1_ − C_i_) × (t_i_ − t_i-1_)/−ln(C_i_/C_i-1_) (3)

CL = A/AUC_0-∞_ (4)

V_d_ = A/C_0_ (5)

K_e_ = In(C_i-1_) − In(Ci)/(t_i_ − t_i-1_) (6)

t_1/2_ = 0.693/K_e_ (7)

where ΔAUC is the area under the curve between two consecutive periods, the AUC_0-∞_ is the area under the concentration–time from 0 to infinity (0 to ∞); C_0_ is the initial concentration of Hex in plasma; A is the injection amount of Hex; t_i_ is the period at Hex concentrations of C_i_; t_i-1_ is the period at Hex concentration of C_i-1_; K_e_ is the constant of the elimination rate.

### 2.7. Molecular Docking

The structures of the ligands S-(+)- and R-(−)-Hex were obtained from the database of Chemicalbook (https://www.chemicalbook.com (accessed on 29 January 2023)). The crystal structures of four P450arom isoforms with PDB IDs of 3EQM, 5JL6, 5JL7, and 5JL9 were provided from the Protein Data Bank (http://www.rcsb.org/ (accessed on 29 January 2023)). The molecular docking of the P450arom isoforms and Hex enantiomers was carried out by the software of Autodock 4.0 (The Scripps Research Institute, San Diego, CA, USA), and the affinity was generated by AutoGrid. The ligand structures were built and minimized by using Discovery Studio software (Accelrys Software Inc., San Diego, CA, USA). Before docking, the original small molecule ligand was removed, the protein was hydrogenated, and its charge was calculated. The molecular docking lattice was set as 40 × 40 × 40 in dimension with spacing of 0.375 angstrom. The Lamarckian genetic algorithm (LGA) was used in the ligand conformation search process. Each molecule performed 50 independent docking operations, and the maximum number of energy assessments was 2.5 million. The other parameters were kept as the default values. The successful prediction was obtained at the condition of the root mean square deviation (RMSD) less than 2 Å. 

### 2.8. Data Analysis

The plasma concentration–time curve data of the Hex were obtained by the DAS 3.0 (Drugs and Statistics, Ver3.0) software. The two-compartment model and related parameters were used to evaluate the toxicokinetic characteristics of the Hex enantiomers. All the obtained data were analyzed by SPSS 17.0 and expressed as the mean ± standard deviation (SD). The t-test was used to compare the parameters of the toxicokinetics for each group. *p* < 0.05 was considered a statistically significant difference.

## 3. Results and Discussion 

### 3.1. Method Validation

A UPLC–MS/MS method for the determination of Hex enantiomers in the plasma, urine, feces, and six tissues of mice was developed. The parameters of method validation, which were recovery, matrix effect, linearity, accuracy, precision, and stability of this method, had been evaluated, and all these results satisfied the determination requirement.

#### 3.1.1. Selectivity 

The chromatograms of the Hex enantiomers were separated by Lux Cellulose-2 chiral columns, which are shown in Figure S2A. The two enantiomers of the Hex were baseline separated with the retention time of 13.02 min and 16.56 min. No interference was observed at the retention time. The chromatograms of pure enantiomers S-(+)- and R-(−)-Hex with the concentration of 1 mg/L are shown in Figure S2B,C. The retention time of these two pure enantiomers was 13.01 and 16.59 min for the S-(+)- and R-(−)-Hex, respectively. No peak was observed at a corresponding time, which demonstrated that the purity of these two enantiomers was relatively high. The rotation directions of the two enantiomers were carried out on a circular dichroism spectrometer (JASCO, Japan), which showed that the first elution was S-(+)-Hex and the second was R-(−)-Hex (Figure S2D,E). 

#### 3.1.2. Recovery and Matrix Effect 

The recovery rates of the Hex enantiomers in the plasma, urine, feces, and six tissues ranged from 88.7% to 104.2%, with RSDs of 2.45–9.45%. The matrix effects of the Hex in different matrices were evaluated by using the ratios of the matrix-spiked peak area to the solvent (acetonitrile)-spiked standard peak area at the levels of 10, 100, and 1000 μg/L with five replicates each. The matrix effects range was 89.6–98.3% for the plasma, 92.3–97.2% for the urine, 93.7–102.3% for the feces, and 90.7–101.5% for the six tissues. No significant matrix effects were observed. 

#### 3.1.3. Linearity

The solvent- and matrix-matched calibration curves were generated at the concentrations ranging from 10 to 1000 μg/L. The linear equations were y = 5637.4x − 12,305 and y = 5817.9.4x − 32,574, and good linearity was achieved with an R2 of 0.9995 and 0.9997 for S-(+)- and R-(−)-Hex, respectively. The matrix-matched calibration curves were used to quantify the real samples to reduce the matrix effects. The limit of detection (LOD) was defined as a signal-to-noise ratio of 3, which was 0.10–0.20 μg/L, and the limit of quantitation (LOQ) was defined as a signal-to-noise ratio of 10, which was 0.50–0.70 μg/L for the Hex enantiomers (Table 1). 

#### 3.1.4. Accuracy and Precision

The accuracy of this method was calculated by comparing the fortified concentrations to the detected concentrations. RSDs were used to evaluate the inter- and intra-day precisions. The inter- and intra-day accuracies were 92.5–95.8%, 90.7–99.3%, 89.4–97.4%, and 88.2–103.4%, with precisions of 0.7–5.6%, 2.45–8.93%, 3.12–9.34%, and 1.23–11.56% for the plasma, urine, feces, and six tissues, respectively. These results showed that the established method was accurate and reproducible for determining Hex enantiomers in mice plasma, urine, feces, and six tissues.

### 3.2. Enantioselective Distributions in Plasma 

**Figure 2:**
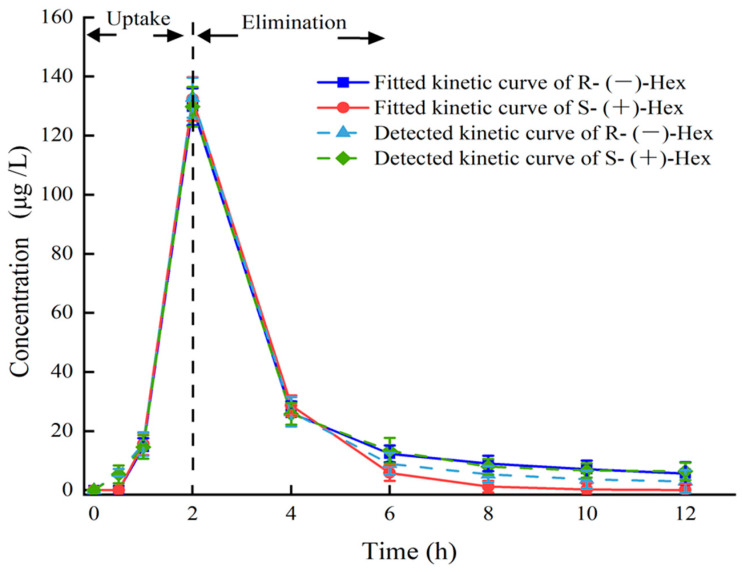
Plasma concentration–time curves of Hex enantiomers after single oral administration at the concentration of 200 μg/L (n = 6).

The established UPLC–MS/MS method was applied to study the toxicokinetics of Hex enantiomers in mice after a single oral administration of Rac-Hex. The fitted kinetic curves and the detected kinetic curves of the Hex enantiomers in the plasma are shown in Figure 2. No significant differences were observed, especially within 5 h, indicating that the kinetic curves were suitable for evaluating the behavior of Hex enantiomers in plasma. The concentrations of S-(+)- and R-(−)-Hex in the plasma after the exposure period of 0, 0.5, 1, 2, 4, 6, 8, 10, and 12 h were detected. The concentrations of S-(+)- and R-(−)-Hex were gradually increased from 5.93 to 132.78 μg/L and from 5.36 to 129.73 μg/L within 2 h, then decreased to 2.92 and 6.37 μg/L in the next 10 h, respectively. The Hex enantiomers reached their highest concentration within 2 h, indicating that the Hex could be absorbed quickly by mice, which was consistent with former reports. The relatively shorter half-life (t_1/2_) and lower maximum concentration (T_max_) indicate that the Hex enantiomers were quickly metabolized, which may pose a low risk to humans. The concentrations of S-(+)- and R-(−)-Hex were below detectable limits after 12 h. The S-(+)-Hex decreased faster in the plasma within the first 6 h, while slower after that compared to its antipode. Significant differences between the two enantiomers were observed (*p* < 0.05). In plasma, the EF values gradually decreased from 1.11 to 1.03 at 4 h and then continued to decrease to 0.46 at 12 h, indicating that R-(−)-Hex was prior to decrease at the first 4 h, while the S-(+)-Hex decreased faster after that period (Figure 4).

The parameters of the toxicokinetics were obtained by fitting the plasma concentration–time curves of the S-(+)- and R-(−)-Hex based on the two-compartment model with the weight coefficient of 1. The C_max_ of the S-(+)- and R-(−)-Hex were 129.73 and 132.78 μg/ L, and the T_max_ were the same at 2 h. The t1/2 of the S-(+)-Hex was 3.07 h, and the R-(−)-Hex was 3.17 h, which was 1.03 times lower than its antipode. The AUC is an important index to evaluate the absorption rate of Hex during the exposure period. The AUC_0–12_ were 304.41 and 321.18 h μg L^−1^ for the S-(+)- and R-(−)-Hex, respectively, indicating that the S-(+)-Hex decreased faster than its antipode. The Vd values were 2.82 and 2.69 L/kg, and the CL of the Hex in plasma were 0.64 and 0.59 L/h/kg for the S-(+)- and the R-(−)-Hex, respectively (Table 2). The relatively high V_d_ and CL values indicated a fast metabolism rate for the S-(+)-Hex. On the other hand, the value of AUC_0-12_ for the R-(−)-Hex was 1.06 times higher than the S-(+)-Hex, accounting for the easier accumulation ability of the R-(−)-Hex. 

### 3.3. Enantioselective Distributions in Six Tissues, Urine, and Feces

**Figure 3:**
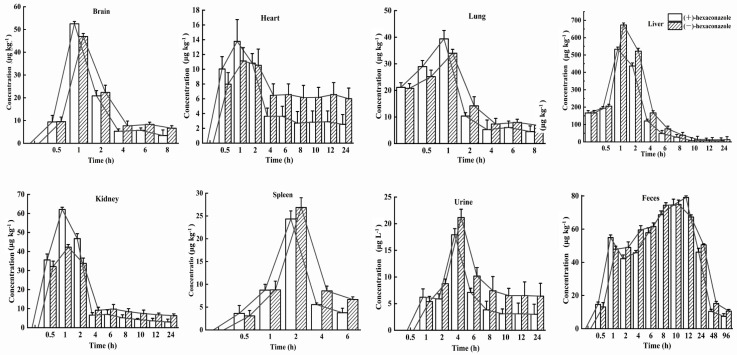
Concentration–time profiles of S-(+)-Hex and R-(−)-Hex in rats after single oral administration at the concentration of 200 μg/kg (n = 6).

The behavior of the absorption, distribution, metabolism, and excretion (ADME) processes of S-(+)- and R-(−)-Hex in six tissues, urine, and feces were determined within 96 h. The Hex enantiomers were distributed rapidly in all the tissues and were detected in all the monitored tissues. However, the concentrations of Hex enantiomers were below the detection limits in most of the tissues within 24 h, except in the feces, which lasted for 96 h. In the spleen, lung, and brain samples, the Hex was detected only in the first 8 h. The distributions of the Hex in the mice followed the order: liver > feces > kidney > brain > lung > spleen > urine > heart. The main elimination organs were the liver and kidney, and the main excretion places were urine and feces. The S-(+)-Hex and R-(−)-Hex reached their maximum concentrations at 1 h in the brain, heart, lung, liver, and kidney, while, in the spleen, urine, and feces, they were 2, 4, and 12 h, respectively (Figure 3). The accumulation ability of exogenous chemicals in the body is mainly related to blood flow speed, hepatic circulation, and chemical properties, as well as gender [22,23].

In the liver, the concentrations of S-(+)- and R-(−)-Hex were detected at 0 h, which were 161.18 and 166.57 μg/kg, then increased to the highest concentrations of 436.66 and 522.04 μg/kg at 2 h, and then decreased to 6.70 and 12.99 μg/kg at 24 h, respectively. In kidney, the concentrations of S-(+)-Hex and R-(−)-Hex were 35.60 and 32.11 μg/kg at 0.5 h and then increased to their highest concentrations 62.10 and 42.29 μg/kg at 1 h and decreased to 3.00 and 6.28 μg/kg at 24 h. After oral administration, most of the Hex accumulated in liver, which is considered as the main detoxification organ and target organ for triazole pesticides in rodents [24]. Penconazole can cause disorders of liver metabolism and alter the gene expression that related to glycolipid metabolism [25]. Meanwhile, Hex has been reported to interfere with tryptophan metabolism in rat hepatocytes in vitro, and activate the kynurenine pathway [26]. Therefore, triazole pesticides could affect liver function in various aspects. On the other hand, they have also been reported to have a toxic effect on the kidney, causing kidney dysfunction [27]. Kidneys are the main excretion organs and the primary target organs of pesticides. Exposure to pesticides can cause rhabdomyolysis, oxidative stress, ischemia-reperfusion injury, and renal tubular epithelial cell damage [28]. Similar results have been reported which showed that the highest concentrations of diisobutyl phthalate were detected in the liver and kidneys in rats, which are the organs that related to metabolism and excretion processes [17]. 

For the brain, the initial concentrations were detected at the first 0.5 h for S-(+)- and R-(−)-Hex, which were 9.38 and 9.53 μg/kg and increased to 52.50 and 46.94 μg/kg at 1 h, and then decreased to 3.42 and 6.63 μg/kg within 8 h. The concentrations of the Hex enantiomers in the brain peaked at 1 h. Relatively high concentrations of Hex were observed in the brain, indicating that S-(+)- and R-(−)-Hex could easily pass through the blood-brain barrier, causing nervous system diseases. Another triazole pesticide, propiconazole, was also reported to damage the nervous system of rats, causing obvious behavioral disorders such as anxiety, depression, and cognitive impairment [29]. Meanwhile, tebuconazole was reported to change the content of neuroprotective factor ceramide in SH-SY5Y neuroblastoma cells, which may have the potential risk of neurodegenerative and Parkinson’s diseases [30]. Although the neurotoxicity caused by Hex has not been systematically studied at present, the detection of its distribution in the brain may indicate its potential risk to the nervous system, which needs further and deeper research.

The initial concentrations of S-(+)- and R-(−)-Hex of 10.04 and 7.98 μg/kg increased to 13.77 and 11.13 μg/kg at 1 h, and then decreased to 2.54 and 6.01 μg/kg at 24 h in the heart. The heart provides sufficient blood flow to the organs and tissues, supplies oxygen and various nutrients, and cleans the end-products of metabolism, such as carbon dioxide, inorganic salts, urea, and uric acid, as well as maintaining the normal metabolism and function of cells. In this study, Hex peaked in the heart at 1 h and fell below the detectable level within 24 h, indicating its persistence in heart. The long-term accumulation period may have a negative impact on the heart function of mice. Tebuconazole was reported to induce oxidative damage by changing the enzyme activities of malondialdehyde (MDA), superoxide dismutase (SOD), catalase (CAT), and glutathione (GSH), and causing cytoplasmic vacuolation, inflammatory cell infiltration, and myocardial disorder in rat hearts [31]. Although relatively low concentrations of Hex were detected in the heart compared to other organs, its 24 h existence could still cause harmful effects on the heart. The concentrations of S-(+)- and R-(−)-Hex were 21.15 and 20.80 μg/kg at 0 h, then increased to their highest concentrations of 39.36 and 33.93 μg/kg at 1 h, and gradually decreased to 4.48 μg/g and 6.79 μg/kg at 8 h for the lung. In the spleen, the Hex was detected within the first 6 h, from 3.63 to 24.34 μg kg^−1^ at 2 h, and then decreased to 3.77 μg/kg for S-(+)-Hex and from 3.08 to 26.86 μg/kg at 2 hm and then decreased to 6.68 μg/kg for R-(−)-Hex. Hex was detected in the lung and spleen in the first 8 h and 6 h with a relatively low concentration and short residence time, indicating a lower potential risk to these two organs.

The main excretion channel for the Hex was feces, followed by urine. In the urine, the concentrations of S-(+)- and R-(−)-Hex were 6.20 and 5.36 μg/L 1 h after oral administration and increased to 17.91 and 21.14 μg/L at 4 h. After that, the concentrations of these two enantiomers decreased to 3.00 and 6.40 μg/kg in 24 h, and then below the detection limit. In the feces, the concentrations of S-(+)-Hex increased from 14.62 to 79.07 μg/kg in the first 12 h, and then decreased to 7.42 μg/kg at 96 h. For the R-(−)-Hex, the concentrations increased from 13.11 to 74.94 μg/kg in 10 h, and then decreased to 10.60 μg/kg^−^ at 96 h. Compared to the urine, the feces achieved a higher concentration and long residence time, indicating that feces were the major excretion channel for Hex. The water solubility of Hex is 0.018 mg/L (20 °C) with a logP value of 3.9, which indicates that the hydrophilicity of Hex is relatively low. This might be the reason that urine is not the main excretion channel for Hex. Although the Hex enantiomers could be detected in the feces within 96 h, most of the Hex was observed at 24 h, which was consistent with the fact that the Hex decreased in all the organs and tissues within 24 h and was excreted through the feces. 

### 3.4. Enantioselectivity in Toxicokinetic Study

**Figure 4:**
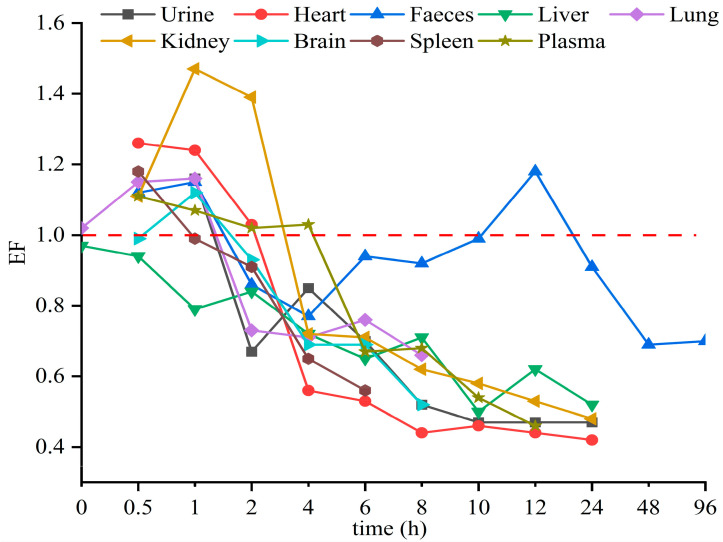
The average EF values of Hex enantiomers in mice after single oral administration at the concentration of 200 μg/kg (n = 6).

The stereoselective metabolism of Hex enantiomers in various tissues was observed in this study. The EF value was introduced to evaluate the stereoselective behavior of the Hex enantiomers in a toxicokinetics study. EF > 1 indicates the R-(−)-Hex was first to decrease, while EF < 1 means the S-(+)-Hex declined faster. As shown in Figure 4, the EF values in the liver were lower than 1 through the whole sampling period, indicating that the S-(+)-Hex decreased faster than the R-(−)-Hex in the liver. In urine and feces, the EF > 1 within the first 2 h of the exposure period and then gradually decreasing below 1, indicated that the (+)-Hex decreased faster first, and then slower, than its antipode after that. The differences in metabolic behavior may be due to the different the protein binding affinity between two enantiomers and proteins [14,32]. 

Triazole pesticides have also shown stereoselective behavior in other mammals and amphibians. For example, R-(−)-Hex has been reported to accumulate in zebrafish more easily than its antipode [15]. In earthworms, the accumulation of S-(+)-Hex takes precedence over that of R-(−)-Hex [8]. However, in rat hepatocytes, the EF values of Hex were 0.53 and 0.91 at 1 h and 24 h, respectively, indicating that S-(+)-Hex was prior to decline [24]. In these reports, the (+)-Hex decreased faster, resulting in the remaining of the (−)-Hex, which was partly consistent with our results. Other triazole pesticides were also reported to achieve enantioselective behavior in different organisms. S-tebuconazole was rapidly metabolized in the plasma and liver of rabbits, and S, S-triadimefon was the first to decline in tadpoles [33,34]. In vitro, stereoselectivity in metabolic behavior of four representative triazole fungicides—prothioconazole, flutriafol, triticonazole, and epoxiconazole—have been observed in rat liver, due to the specific affinity of CYP enzymes in the liver [35,36]. All in all, the enantiomer behavior of triazole pesticides differed due to various reasons, while the most important one is the binding affinity between enantiomers and enzymes. 

### 3.5. Molecular Docking of Hex to P450arom

Based on the results of stereoselectivity in the toxicokinetics study of Hex enantiomers in rats, molecular docking was performed to mechanistically reveal the differences in metabolic behavior caused by the Hex enantiomers. The interactions between the Hex enantiomers and P450arom were performed by molecular docking. The parameter of binding energy was used to predict the metabolic capacity. The lower the energy, the faster the Hex metabolism. The results of the docking showed that the isoforms of 3EQM showed the lowest binding energy to S-(+)-Hex, which was −6.47 kcal/mol, and to R-(−)-Hex was −5.72 kcal/mol, followed by 5JL7, with binding energies of −6.02 and −5.84 kcal/mol, 5JL9 of −5.95 and −5.50 kcal/mol, and 5JL6 of −5.83 and −5.81 kcal/mol for S-(+)-Hex and R-(−)-Hex, respectively. These results indicate that 3EQM showed the strongest binding affinity with Hex enantiomers. The binding energy of S-(+)-Hex to four isoforms of P450arom were all lower than R-(−)-Hex, indicating that the S-(+)-Hex combined much more stable with P450arom and exhibited higher metabolic rate which was consistent with the results of toxicokinetic with S-(+)-Hex metabolized faster than its antipode in most tissues. 

**Figure 5:**
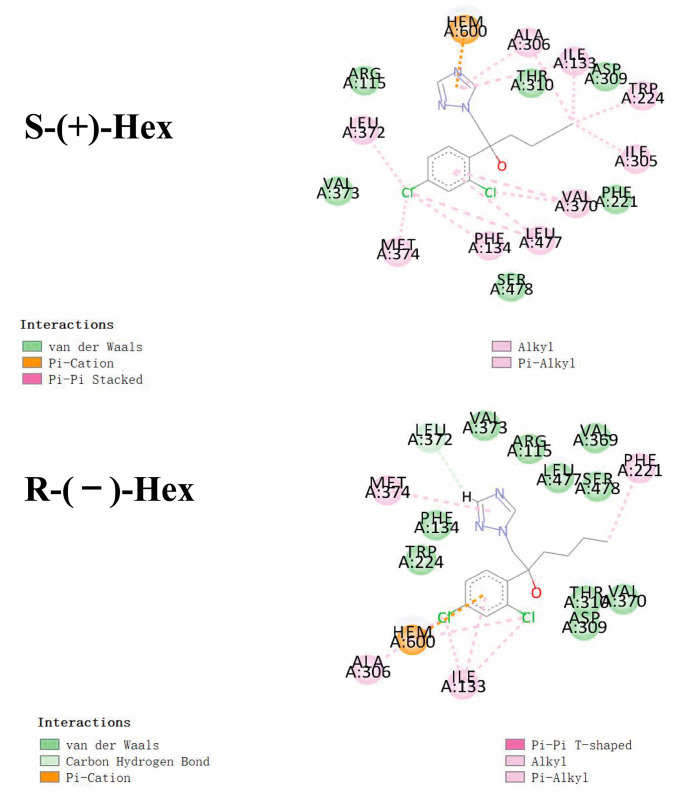
Molecular docking conformations for S-(+)-Hex and R-(−)-Hex with P450arom isoforms of 3EQM.

As shown in Figure 5, Hex mainly binds to the amino acids of VAL (A:370, A:373), LEU (A:477, A:372), and PHE (A:134, A:221). Similar interacted amino acids were detected after docking with the two enantiomers. However, little difference was observed with VAL A:369, which was only observed in the R-(−)-Hex, while ILE A305 was bind with S-(+)-Hex only. The main interaction bond types of the S-(+)-Hex with the 3EQM were Alkyl and Pi-Alkyl, while for the R-(−)-Hex, the main bond type was van der Waals. This might explain the reason that the S-(+)-Hex combined much more stably with the 3EQM than did its antipode. The docking results of the other three P450arom isoforms, 5JL7, 5JL9, and 5JL6, were similar to the 3EQM (Figure S3). These results showed that the differences in metabolic behavior of the Hex enantiomers were mainly due to the types of interaction bond. Reports have shown that S-(+)-Hex had higher acute toxicity in earthworms and achieved higher toxicity on cytochrome P450 than R-(−)-Hex [8]. The hepatic microsomal enzymes of mice preferred to degrade the S-(+)-Hex than its antipode [14]. The S-(+)-Hex was the first to decline in rat hepatocytes, and significantly affected the level of tryptophan metabolism [25]. These results were consistent with the results of our study, and the molecular docking explained the reasons at the molecular mechanism level. 

## 4. Conclusions

In this study, a UPLC–MS/MS method was established to monitor the distribution of Hex enantiomers in mice, and molecular docking was used to reveal the differences in metabolic behavior of the Hex enantiomers at mechanistical levels. Under the established method, two enantiomers of Hex were well separated with good accuracy and precision, and high sensitivity was achieved, which indicated its reliability for quantification. This method was then used to evaluate the toxicokinetics of Hex enantiomers in mice. The results showed that the Hex was mainly distributed in the liver, kidney, and excreted mainly by the feces. A stereoselectivity in the ADME processes was observed in all the monitored organs and tissues, indicating the differences in the potential toxic risks. The results of molecular docking showed that the binding energy of S-(+)-Hex with P450arom was lower than its antipode, indicating its higher metabolic rate, which illustrated the reason that the S-(+)-Hex decreased faster in most of the tissues or organs. The results of this study could improve the understanding of the toxic effects and potential risks of Hex at the enantiomer level and guide its suitable usage in agriculture.

## Figures and Tables

**Table 1:** Calibration curves, coefficient (R^2^), limit of detection (LOD), and limit of quantification of Hex enantiomers (LOQ) (n = 3).

Samples	Enantiomers	Calibration Curves	R^2^	LOD (μg L^−1^)	LOQ (μg L^−1^)	Recoveryrate (%)	RSDs (%)
Plasma	S-(+)-Hex	y = 5951x − 64555	0.9956	0.20	0.70	90.3	3.45
	R-(−)-Hex	y = 5889x − 58921	0.9945	0.20	0.70	89.2	4.56
Urine	S-(+)-Hex	y = 6323x − 45432	0.9957	0.10	0.60	91.4	7.58
	R-(−)-Hex	y = 6454x − 34324	0.9945	0.10	0.60	90.3	4.54
Feces	S-(+)-Hex	y = 6345x − 54354	0.9912	0.10	0.60	101.3	6.41
	R-(−)-Hex	y = 6534x − 87645	0.9945	0.10	0.60	104.2	8.46
Heart	S-(+)-Hex	y = 5645x − 79521	0.9972	0.10	0.50	94.6	3.56
	R-(−)-Hex	y = 5956x − 74413	0.9986	0.10	0.50	88.7	5.79
Liver	S-(+)-Hex	y = 6721x − 56843	0.9945	0.10	0.50	96.3	6.75
	R-(−)-Hex	y = 6942x − 52485	0.9978	0.10	0.50	98.5	4.56
Lung	S-(+)-Hex	y = 7842x − 45214	0.9996	0.10	0.50	89.2	7.87
	R-(−)-Hex	y = 7254x − 49511	0.9986	0.10	0.50	88.8	9.46
Kidney	S-(+)-Hex	y = 5952x − 22457	0.9982	0.10	0.60	101.4	3.43
	R-(−)-Hex	y = 5142x − 55621	0.9945	0.10	0.60	103.5	2.51
Brain	S-(+)-Hex	y = 4158x − 75121	0.9998	0.10	0.50	91.3	5.49
	R-(−)-Hex	y = 4682x − 84520	0.9976	0.10	0.50	94.6	7.59
Spleen	S-(+)-Hex	y = 8614x − 42152	0.9946	0.10	0.50	98.3	9.45
	R-(−)-Hex	y = 8145x − 46212	0.9978	0.10	0.50	99.5	8.68

**Table 2:** Pharmacokinetic parameters of S-(+)- and R-(−)-Hex in plasma after oral administration of Rac-Hex (n = 6).

Pharmacokinetic Parameters	Parameter Values		Unit
	S-(+)-Hex	R-(−)-Hex	
AUC_0–12_	304.41 ± 20.12	321.18 ± 19.11	µg/L × h
AUC_0-∞_	314.38 ± 12.11	339.99 ± 11.12	µg/L × h
t_1/2_	3.07 ± 1.13	3.17 ± 0.78	h
T_max_	2.00 ± 0.28	2.00 ± 0.55	h
Vd	2.82 ± 1.12	2.69 ± 0.91	L/kg
CL	0.64 ± 0.09	0.59 ± 0.05	L/h/kg
C_max_	132.78 ± 13.14	129.73 ± 10.12	µg/L

## Data Availability

Not applicable.

## Supplementary Materials

The following supporting information can be downloaded at: https://www.mdpi.com/article/10.3390/toxics11020145/s1, 2.5. Method validation: Figure S1: Two-compartment model of oral administration; Figure S2: The chromatograms and rotation directions of Hex enantiomers; Figure S3: Molecular docking conformations of S-(+)-Hex (A) and R-(−)-Hex (B) with P450arom isoforms of 5JL6, 5JL7, and 5JL8.
