# Supplementary material for: Stereoselective Toxicokinetic and Distribution Study on the Hexaconazole Enantiomers in Mice
Source: Toxics. 2023 Feb 2;11(2):145. doi: 10.3390/toxics11020145 (PMC9966998; doi:10.3390/toxics11020145)
Supplement: Supplementary file 1 [file toxics-11-00145-s001.zip › toxics-2103597-supplementary.pdf]

Supplementary Information

Stereoselective toxicokinetic and distribution study on the hexaconazole enantiomers in mice

Guofei Luo[1], Junxiao Pang[2], Dali Sun[1,*], Qinghai Zhang[1,*]

[1] School of Public Health/the Key Laboratory of Environmental Pollution Monitoring and Disease Control, Ministry of Education, Guizhou Medical University, Guiyang, 550025, China

[2] Food and Pharmaceutical Engineering Institute, Guiyang University, Guiyang, 550005, China [*] Corresponding author: Dali Sun and Qinghai Zhang

2.5 Method validation

The stock solution of Rac-Hex (1 mg/mL) was serially diluted in acetonitrile and standard solutions were obtained as 10, 50, 100, 200, 500, 1000 μg/L. The matrix standard solutions were prepared by diluting the stock solution with blank plasma, urine, feces and 6 tissues extraction instead of acetonitrile. The above mentioned 9 blank samples were added with Rac-Hex standard and generated the fortified levels of 10, 100, 1000 μg/L with 5 replicates each. Samples were then prepared and detected by following the methods of section 2.3 and 2.4. Recovery rate was determined by the ratio of peak areas between the extracted analyte and the matrix-matched standard solution. Matrix effects were estimated by the ratio of matrix standard peak areas to solvent standard peak areas at the same concentration. The inter- and intra-day accuracy and precision were conducted by fortifying the blank extracts of plasma, urine, feces, and 6 tissues with Rac-Hex standards at the levels of 10, 100 and 1000 μg/L within 1 day and 3 days consecutive injection. The limit of detection (LOD) was defined as the concentration that generated a signal-to-noise (S/N) ratio of 3, and the limit of quantification (LOQ) was defined as the lowest concentration in the calibration curve with acceptable accuracy and precision. Three concentrations of matrix standards were mixed and used as quality control (QC) samples which were injected every 10 samples.

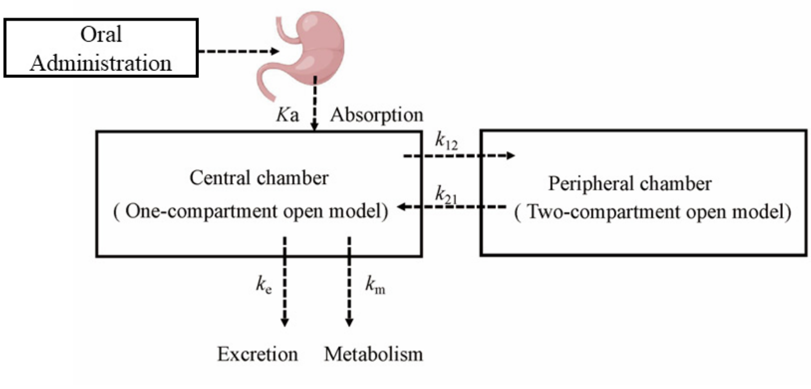

Figure S1. Two-compartment model of oral administration.

K: constant of absorption rate; K_{12}, K_{21}: the rate constant of Hex transport from the first compartment to the second compartment (K_{12}) and from the second compartment to the first compartment (K_{21}); *Km*, *Ke* is the rate constant of metabolism and excretion, respectively.

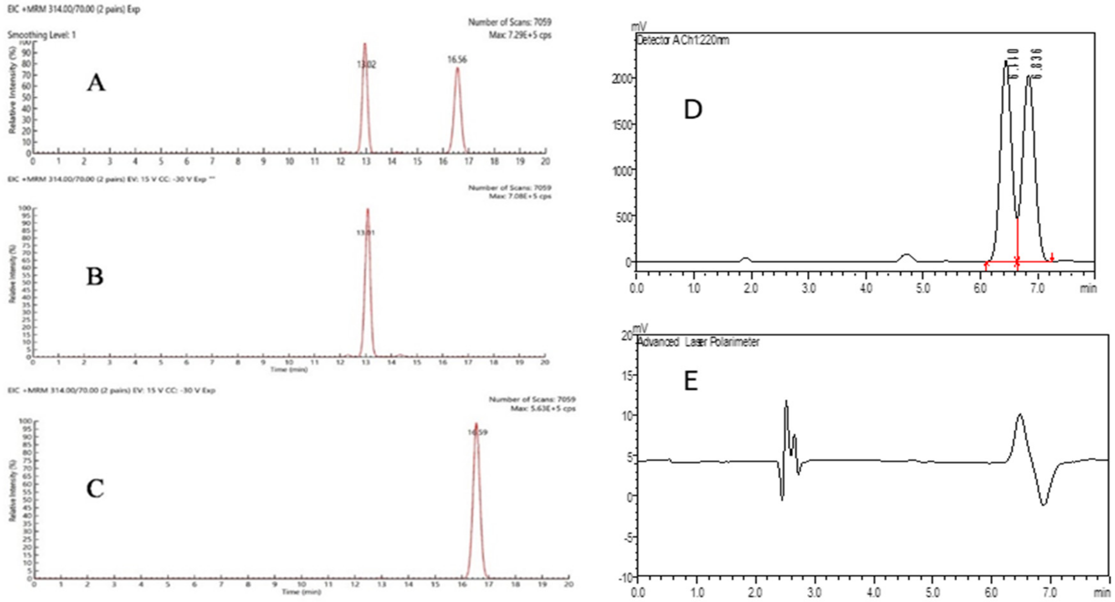

Figure S2. The chromatograms and rotation directions of Hex enantiomers.

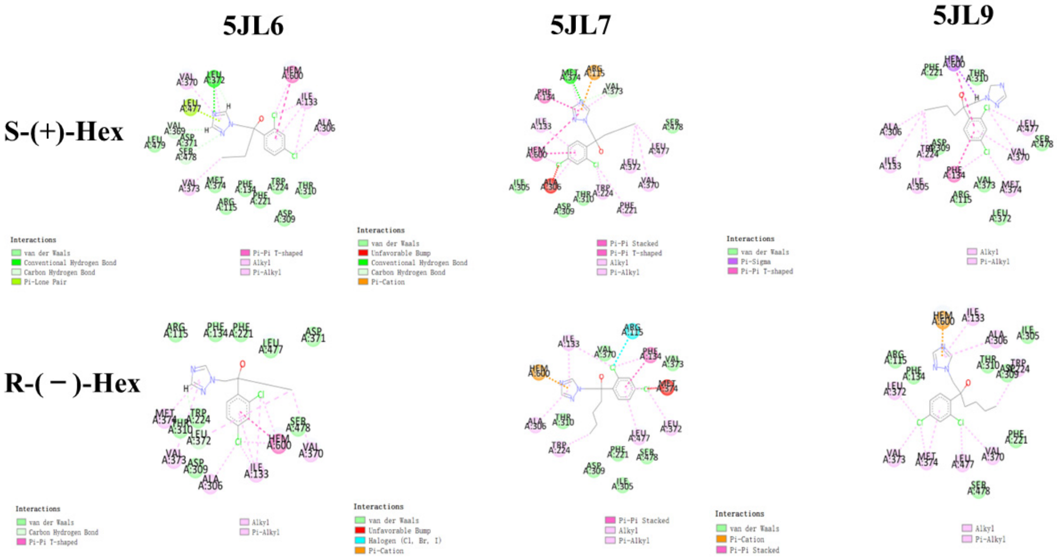

Figure S3. Molecular docking conformations of S-(+)-Hex (A) and R-(−)-Hex (B) with P450arom isoforms of 5JL6, 5JL7, and 5JL8.
